# Descriptive Epidemiology of Gastric Cancer: A Population-Based Study From Georgia

**DOI:** 10.7759/cureus.66862

**Published:** 2024-08-14

**Authors:** Saba Zhizhilashvili, Irakli Mchedlishvili, Rolando Camacho, Natalia Jankarashvili, Natalia Garuchava, Nana Mebonia

**Affiliations:** 1 Epidemiology and Biostatistics, Tbilisi State Medical University, Tbilisi, GEO; 2 Global Technical Advisor, City Cancer Challenge Foundation, Geneva, CHE; 3 Oncology, World Health Organization, Mallorca, ESP; 4 Radiation Oncology, F. Todua Medical Center, Tbilisi, GEO

**Keywords:** incidence, georgia, descriptive epidemiology, gastric cancer, cancer registries

## Abstract

Background

Gastric cancer (GC) remains a significant public health issue in many countries globally due to its high morbidity and mortality rates. In Georgia, the incidence of GC reflects the prevalence patterns of established risk factors. To develop appropriate prevention and treatment strategies, GC requires a comprehensive approach and research. This study aims to review and describe GC epidemiologic characteristics in the country.

Methodology

We conducted a descriptive analysis utilizing data from the national population-based cancer registry. All patients diagnosed with invasive GC between 2015 and 2022 were eligible for inclusion in the analysis. To calculate age-standardized incidence (ASIR) and mortality (ASMR) rates we used a direct method, standardized to the World (WHO 2000-2025) standard population. Trends in Incidence and mortality were assessed using standardized rate ratios (SRRs). The mortality-to-incidence ratio (MIR) was defined as the ratio of the ASMR to the ASIR for the corresponding year. The Kaplan-Meier method was utilized to construct survival curves with survival comparisons performed using the log-rank test.

Results

A total of 2,707 GC cases with 62% (n = 1,668) of patients being male were enrolled in this descriptive study. The median age at diagnosis was 65 years, and about 70% (n = 1,893) of cases were detected at advanced (III and IV) stages. Over the study period, the ASIR per 100,000 population for both sexes decreased from 8.4 to 7.3. The SRR and 95% confidence interval indicated no significant change in ASIR for males but it decreased for females in 2022 compared to 2015. In 2022, the ASMR decreased compared to 2015 for males (from 10.5 to 7.3/100,00) and for females (from 5.8 to 3.0/100,000) as well. However, the MIR ​​indicated an unstable reduction in mortality, fluctuating over the observation period. The five-year survival rate was around 22.0%.

Conclusions

This study provides a comprehensive overview of GC epidemiology in Georgia between 2015 and 2022. GC remains a significant public health challenge, characterized by the high proportion of late-stage diagnoses and high mortality rates. The implementation of prevention and early diagnosis strategies is crucial to reduce the burden of GC in the country.

## Introduction

Gastric cancer (GC) remains an important public health issue in many countries globally due to its high morbidity and mortality. It was the fifth most frequently diagnosed malignant tumor and the fourth leading cause of cancer deaths worldwide in 2020 [1,2]. Despite the achievements in cancer management, the prognosis of GC continues to be poor, with a five-year survival rate of around 20% [3].

The burden of GC varies throughout regions, with Asia reporting the highest incidence rates, followed by Eastern and Central Europe [4-8]. High-incidence countries prioritize prevention and early detection of disease. GC is caused by various modifiable risk factors, including *Helicobacter pylori* infection, high intake of salty and smoked food, tobacco smoking, harmful use of alcohol, low physical activity, and low consumption of fruits and vegetables, allowing for primary prevention of disease [9-11]. The early detection of GC through screening is another prevention activity, which is recommended for high-risk groups [3,12].

In Georgia, between 2015 and 2019, according to the population-based cancer registry database, GC ranked among the top five most frequently diagnosed cancers, along with breast, lung, prostate, and thyroid cancer [13].

The high incidence of GC reflects the prevalence patterns of its well-established risk factors in the country. Studies have shown that 37% of the population are smokers in Georgia [14]; harmful use of alcohol (according to the World Health Organization definition of >40 g for women and >60 g for men of pure alcohol) slightly exceeds 10%; and about 18% of both sexes engage in heavy episodic drinking (taking six or more standard doses even on one day in the last 30 days) [15]. Furthermore, a large share of the Georgian population does not follow healthy diet recommendations, which is proved by several indicators. Fruit and vegetable consumption is low, with nearly two-thirds of both sexes consuming less than 400 g of fruit and vegetables daily. The population’s awareness about the harmful effects of salt is high, with almost 73% of men and 83% of women believing that salt can harm health. However, more than a quarter of the population (27%) always or often add salt or salty sauces to their meals before or during food consumption. Consequently, 64.6% of adults are overweight or obese, highlighting the broader health challenges contributing to GC risk [16].

The three-year overall survival rate for GC patients diagnosed between 2015 and 2019 was 30.2% in Georgia [13]. To improve survival through the development of appropriate prevention strategies and treatment approaches, the disease patterns require comprehensive research. This study aims to review and describe GC epidemiologic characteristics in Georgia.

## Materials and methods

Study setting and design

To conduct a descriptive analysis, data on GC cases were extracted from the Georgian population-based cancer registry database. The study protocol was approved by the Ethics Committee of Tbilisi State Medical University, Georgia. According to the study protocol, we used only non-personalized data to perform statistical analysis; therefore, a written informed consent form was not required.

Study participants

We extracted GC cases from the National Population-based Cancer Registry, which has been collecting data on newly diagnosed cancer cases since 2015. All patients diagnosed with invasive GC (topography codes C16.0-C16.6 and C16.8-C16.9 in the third edition of the International Classification of Diseases for Oncology, ICD-O-3) between January 1, 2015, and December 31, 2022, were eligible for inclusion in the analysis. Cases with incomplete data (2.5%, n = 67) and those diagnosed after death (3.5%, n = 96) were excluded from the analysis (due to lack of information). The International Agency for Research on Cancer Check Tool is used to monitor the quality of data in the Georgian cancer registry. In addition, the quality of data is cross-checked and completed by comparing it with independent sources, such as hospital discharge registries, social care agency databases, and national mortality databases. To validate the cancer mortality data, we requested information from the National Statistics Office of Georgia (GeoStat, https://www.geostat.ge/en), which operates the mortality database, and compared it with the cancer registry database. In addition, as the GeoStat monitors the population and demographic statistics, population numbers by calendar year, stratified by sex and age groups were obtained from this source.

Study variables

The following variables were obtained for statistical analysis: sex, age at diagnosis, stage at diagnosis, calendar year of diagnosis, and calendar year of death. Age at diagnosis was divided into 12 five-year interval age groups and ≥85 years, while the stage of cancer at diagnosis was divided into five groups (I, II, III, IV, and unknown).

Study endpoint

The observation period for study participants covered the time from the date of diagnosis until the end of the study (August 6, 2023) for surviving patients, or until the date of death for those who deceased during follow-up. The linkage between the cancer registry and the national mortality database allows for up-to-date information on the vital status of cancer patients. The overall mortality of GC patients, including all-cause mortality, was considered the study endpoint.

Statistical analysis

Incidence and mortality rates were stratified by sex and age groups. We used a direct method to calculate age-standardized incidence (ASIR) and age-standardized mortality (ASMR) rates per 100,000 person-years. Rates were standardized to the World (WHO 2000-2025) standard population [17].

The mortality-to-incidence ratio (MIR) was defined as the ratio of the ASMR to the ASIR for the corresponding year. The MIR compares the number of deaths from disease with the number of newly diagnosed cases in a given calendar year; an MIR of 1 indicates an equal number of deaths and newly diagnosed cases, while an MIR less than 1 shows fewer number of deaths, but an MIR greater than 1 highlights a higher number of deaths from disease than were diagnosed in a given year. Therefore, decreasing MIR over time could reflect improvements in survival (decreasing mortality), improved early diagnoses (increasing recorded incidence), or a combination of both.

We estimated the trends in incidence and mortality by calculating the standardized rate ratios (SRRs), which were defined by dividing the ASIR or ASMR of the most recent period (2020) by the ASIR or ASMR of the earliest period (2015). An SRR greater or less than 1.0 with non-overlapping 95% confidence intervals (CIs) was considered to indicate increasing or decreasing trends, respectively. An SRR with 95% CIs including 1.0 was considered to be stable.

Age-specific incidence rates were calculated per 100,000 individuals using five-year age groups (15-19; 20-24; 25-29; 30-34; 35-39; 40-44; 45-49; 50-54; 55-59; 60-64; 65-69; 70-74 and ≥85). To calculate age-specific incidence rates for the period 2015 and 2022, the total number of new cases in a specific age group was divided by the total number of person-years in the same age group. The person-years for every specific age group and sex by calendar years were taken from the GeoStat.

In analytical statistics, the Kaplan-Meier method was utilized to construct survival curves with survival comparisons performed using the log-rank test. P-values of <0.05 were counted as statistically significant. Obtained data were analyzed using SPSS version 23 (IBM Corp., Armonk, NY, USA).

## Results

Characteristics of gastric cancer patients

A total of 2,707 GC cases, diagnosed between 2015 and 2022 in Georgia and registered in the national population-based cancer registry, were enrolled in a descriptive study. The median age at diagnosis was 65 years (age range: 15-96), with 62% (n = 1,668) males. About 70% (n = 1,893) of patients were diagnosed at the advanced (III and IV) stages (Table 1).

**Table 1:** Characteristics of gastric cancer patients enrolled in the analyses, 2015-2022, Georgia (n = 2,707)

Variables	n	%
Sex		
Male	1,668	62.0
Female	1,039	38.0
Age at diagnoses		
Median (age range)	65 years	(15–96)
Under 20 years	6	0.2
20–29 years	13	0.5
30–39 years	63	2.3
40–49 years	174	6.4
50–59 years	546	20.1
60–69 years	916	33.9
70–79 years	735	27.2
Over 80 years	254	9.4
Stage at diagnosis		
I	98	3.6
II	291	10.7
III	758	28.0
IV	1,135	42.0
unknown	425	15.7

Age-standardized incidence rates

In Georgia between 2015 and 2022, GC was the seventh most frequently diagnosed type of cancer after breast, thyroid, lung, colorectal, prostate, and bladder cancer, while its incidence ranked fifth among males (after lung, prostate, colorectal, and bladder cancer), and seventh among females (after breast, thyroid, colorectal, cervical, corpus uteri, and ovarian cancer). Over the study period, the ASIR per 100,000 population for both sexes decreased from 8.4 to 7.3, with a small variation between these two values. The same trend of a slight decrease in ASIR was observed among males and females, and it changed from 12.0 to 11.5 in males, and from 5.9 to 4.3 in females. In addition, the ASIR for males was about twice as high as for females across all calendar years (2015-2022) included in the analyses (Table 2).

**Table 2:** Gastric cancer age-standardized incidence rates (ASIRs) and age-standardized mortality rates (ASMRs) per 100,000 person-years and mortality-to-incidence ratio (MIR) by sex, Georgia, 2015-2022.

Rates	2015	2016	2017	2018	2019	2020	2021	2022
Both sexes								
ASIR	8.4	7.8	7.5	6.3	6.2	7.4	6.9	7.3
ASMR	7.7	8.1	8.1	8.1	8.0	9.0	7.5	4.8
MIR	0.92	1.04	1.04	1.29	1.29	1.22	1.09	0.66
Males								
ASIR	12.0	12.1	10.3	9.0	9.5	10.4	9.7	11.5
ASMR	10.5	12.3	12.2	12.6	12.3	14.4	12.2	7.3
MIR	0.88	1.02	1.18	1.4	1.29	1.38	1.26	0.63
Females								
ASIR	5.9	4.7	5.7	4.3	3.8	5.1	4.8	4.3
ASMR	5.8	5.2	5.3	5.2	5.0	5.3	4.2	3.0
MIR	0.98	1.11	0.93	1.21	1.32	1.04	0.88	0.70

Age-specific incidence rates

During the study period, no GC cases were registered under the age of 16 years, 4% of cases occurred in individuals aged 15-40 years, while about 70% of cases were detected after the sixth decade of life (Table 1). The age-specific incidence rates increased with aging in both sexes, reaching peak values in the 70-79-year age group for both sexes, males and females (Table 3).

**Table 3:** Age-specific incidence rates per 100,000 individuals for gastric cancer patients by sex, Georgia, 2015-2022.

Age groups (years)	Both sexes	Male	Female
15–19	0.4	0.2	0.5
20–24	0.05	0	0.1
25–29	0.6	0.4	0.8
30–34	1.0	1.1	0.8
35–39	2.1	1.9	2.3
40–44	4.0	4.5	3.6
45–49	5.1	6.4	3.8
50–54	10.2	14.4	6.4
55–59	17.0	29.5	10.1
60–64	25.0	36.0	15.6
65–69	31.1	47.7	19.3
70–74	40.3	63.7	25.4
75–79	36.5	57.0	25.0
80–84	29.0	47.0	19.9
≥85	17.9	29.3	13.2

Age-standardized mortality rates

During the observation period, GC ranked third in cancer mortality, following lung and breast cancers. The ASMR per 100,000 population decreased from 8.4 to 7.3 overall (for both sexes). The indicator followed the same trend for males and females, dropping from 10.5 to 7.3 in males and from 5.8 to 3.0 in females with a slight increase in 2020 (Table 2).

Standardized rate ratios

Table 4 shows time trends in GC incidence, demonstrated by SRRs. The SRRs (for incidence), as explained in the Methodology, is the ratio of incidence rates from the most recent period (2022) to the incidence rates from the earliest period (2015). According to the SRRs and 95% CIs, ASIR for males in 2022 was almost the same as in 2015 but showed a decreasing trend in females (SRR = 0.96; 95% CI = 0.79-1.15 for males vs. SRR = 0.73; 95% CI = 0.53-0.98 for females).

**Table 4:** Gastric cancer standardized incidence rate ratios and standardized mortality rate ratios and 95% CIs by sex, Georgia, 2015-2022. SRR: standardized rate ratio; CI: confidence interval

Sex	SRR	95% CI
SRR for incidence		
Both sexes	0.87	0.68–1.09
Males	0.96	0.79–1.15
Females	0.73	0.53–0.98
SRR for mortality		
Both sexes	0.62	0.46–0.83
Males	0.70	0.55–0.87
Females	0.52	0.35–0.74

The SRRs for overall mortality and 95% CI indicate that it decreased for males and females in 2022 compared to 2015 (SRR = 0.70; 95% CI = 0.55-0.87 for males vs. SRR = 0.52; 95% CI = 0.35-0.74 for females). However, the overlapping 95% CIs suggest non-significant sex differences (Table 4).

Mortality-to-incidence ratio

The MIR is a sensitive indicator, with its values ​​indicating an unstable decline in mortality. It fluctuated over the observation period (2015-2022) in both sexes (Table 2). However, recently (2020-2022), it has been more favorable in women, demonstrating decreasing trends in mortality.

Kaplan-Meier survival analyses

**Figure 1:**
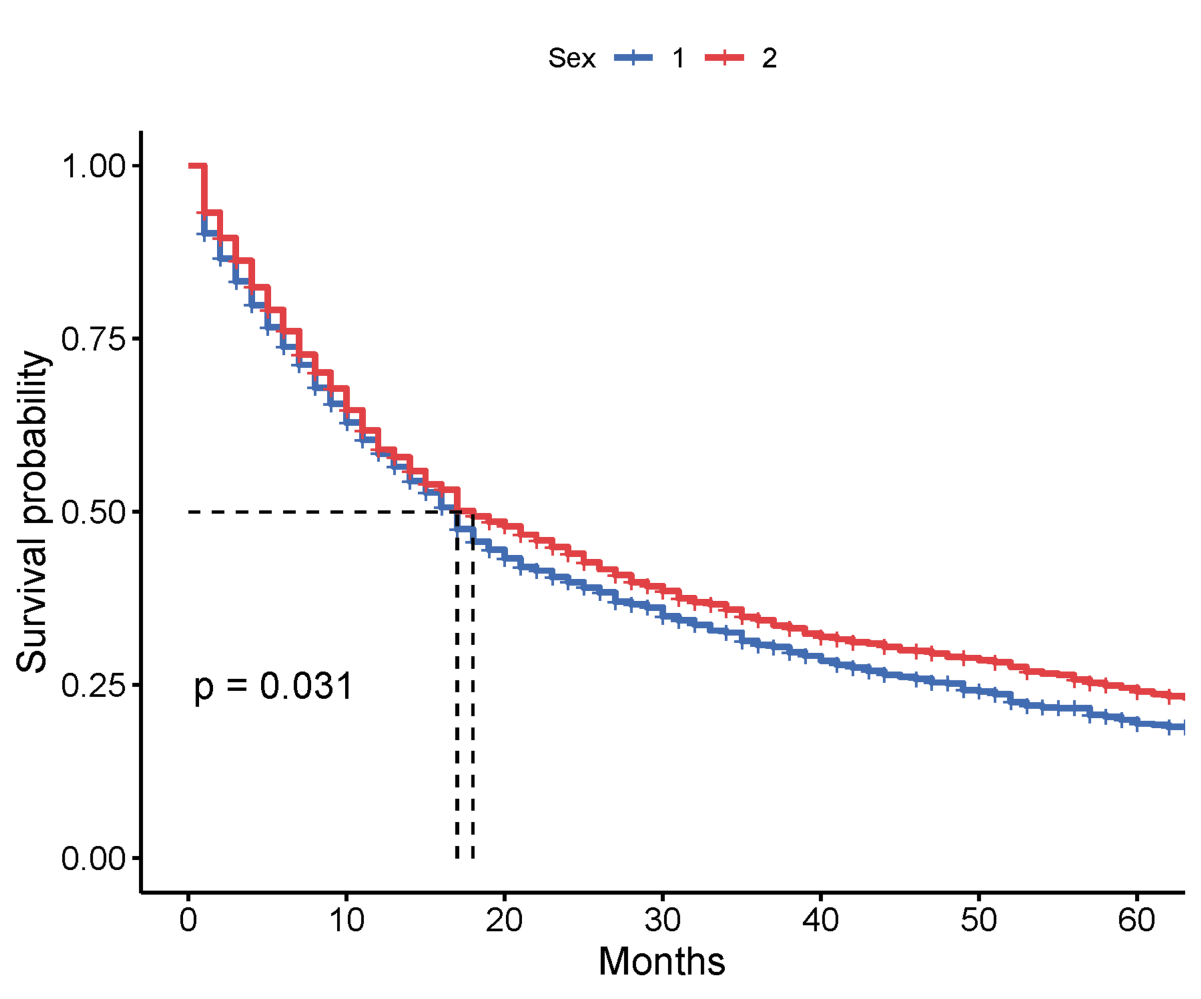
Kaplan-Meier survival analysis by sex of patients. Sex of GS patients: 1: male; 2: female.

A Kaplan-Meier survival analysis by sex for GC patients, diagnosed between 2015 and 2022, revealed a very similar median for overall survival at 17 months (95% CI = 16-18 months) and 18 months (95% CI = 17-22 months), for males and females accordingly (p = 0.031, log-rank test). Five-year survival rates were 22.0% (95% CI = 19.0-25%) for females and 20.0% (95% CI = 18.0-22%) for males (Figure 1).

## Discussion

To estimate the epidemiologic profile of GC in Georgia, we conducted an observational retrospective study, by utilizing the population-based cancer registry data. In total, we analyzed 2,707 GC cases diagnosed and registered in the system between 2015 and 2022. During the study period, GC ranked as the seventh most commonly diagnosed cancer. The burden of GC in Georgia is determined by its high mortality, it is the third leading cause of cancer death in the country. GC Incidence and mortality in men were approximately twice as high as in women in all calendar years studied. The incidence among males remained mainly stable, but in females, it decreased in 2022 compared to 2015. GC mortality analyses demonstrated its high rates, with only an unstable reduction. One of the possible reasons for the high mortality of GC patients could be the late detection of the disease. About 70% of GC cases are diagnosed at advanced (III and IV) stages.

As in Georgia, GC remains one of the leading causes of cancer morbidity and mortality in many countries worldwide [8,18]. However, there is a large global variability in the incidence and mortality rates for GC [1,11,19-23]. Eastern Asia presents the highest incidence of GC for both sexes, males and females. Other high-incidence regions are Eastern and Central Europe and Latin America, while the Incidence remains low in West Europe, North America, and the African region [4-7,21,24]. In high-risk regions, the ASIRs per 100,000 individuals (males and females accordingly) varied between 32.5 and 12.0 among males and between 13.2 and 6.0 among females, respectively, in 2020 [25]. GC mortality varied widely across countries and regions. The highest mortality rates were registered in the Eastern Asia region (14.6 per 100,000 people), followed by Latin America, Western Asia, and Central and Eastern Europe [3]. A lower share of death was observed in very high human development index (HDI) countries compared to medium and low HDI countries [5].

Significant declining trends in the incidence and mortality from GC are the major epidemiological features globally and have been observed consistently in both men and women; however, the mortality rates have decreased more rapidly than incidence [26-30]. Possible reasons for the decrease in the incidence and mortality of GC over the past few decades are multiple, including improvements in diet, food quality, and storage, reduced salt intake, declined prevalence of *H. pylori* infection through improved prevention and treatment approaches, and encouraged early diagnosis programs [3,29,31,32]. Unfortunately, GC incidence and mortality rates revealed by the study are not consistent with international trends and do not show a stable tendency of decline.

The age at diagnoses of GC patients in Georgia mainly corresponds to global patterns. In general, the incidence rate of GC rises gradually with age [7,10,22,31]. The global ASIRs and ASMRs increased linearly with age in females (showing no decreasing trend in elders), while the same indicators increased nonlinearly in males, peaking at ages 85-89 years [10]. The median age of diagnoses and the age groups, when the disease reaches peak values vary greatly (moderately) across regions and countries, and one reason for this variation might be the life expectancies of the populations. In many countries, the highest incidence of GC is observed in the fifth and sixth decades of life, while the risk is very low among individuals younger than 44 years [22]. For example, in the United States, approximately 1% of cases occurred among the age groups of 20-34 years, while 29% of cases were detected between 75 and 84 years of age. The median age at diagnosis of GC was 70 years [33]. At the same time, recent findings indicated an increase in the incidence of GC among young adults aged under 50 years in both low-risk and high-risk countries [34]. Additionally, the literature reviews demonstrate GC diagnoses at younger ages in Africa and Latin America [4].

Sex differences in GC cases revealed in Georgia are consistent with global characteristics. In 2020, 66% of GC new cases diagnosed globally were in males [1,5]. Accordingly, the global ASIR and ASMR were higher in males than females, except for the death rates in those 15-29 years of age [10]. The incidence rates of GC were about two to three times lower in females compared to males across the countries [2,3,22,33]. Additionally, in males, GC was the leading cause of cancer death in several South-Central Asian countries, including Iran, Afghanistan, Turkmenistan, and Kyrgyzstan [25,34]. Lifestyle characteristics related to smoking and diet are considered to contribute to sex differences in GC. However, there is growing evidence that the underlying biological sex features are reasons for these differences. Many studies have supported the crucial role of estrogen receptors in avoiding GC, but it needs further investigation [10].

Early diagnoses of GC continue to pose many challenges in Georgia, as in many other countries globally [2,8,18,22]. As the disease usually progresses asymptomatically or rarely causes specific symptoms, diagnosing GC in the early stages is difficult [2,27]. Outcomes from GC remain poor, the five-year survival ranges between 20% and 40% in most countries [27]. However, in high-risk countries of East Asia, population-based screening programs are implemented, which support improvements in the early detection and survival of GC [3,18].

As the survival rate of GC is strongly stage-dependent, it remains low in Georgia. It was less than 25% during the study period. Many other factors can determine the survival of GC patients, including the type of cancer, the age at diagnosis, underlying conditions, accessibility to medical care, treatment approaches, lifestyle, etc. However, strategies to improve GC prognosis should focus on early diagnoses of disease in Georgia. The country has launched early diagnosis programs aimed at increasing public awareness and the knowledge of general practitioners on the initial symptoms of highly prevalent oncological diseases, although intensification of these activities is necessary [35]. Additionally, the universal health coverage (program, introduced by the government of Georgia in 2013, was expanded in 2023 and covers all types of treatment for cancer patients. Moreover, human resources, needed for GC management, are readily available in the country [35]. However, whether all patients receive the standardized treatment, based on the European Society for Medical Oncologists or the American Society of Clinical Oncology guidelines, needs additional investigation.

A strength of our study is the first comprehensive description of GC epidemiologic characteristics at the national level. Features of the disease stages at diagnosis and long-term trends in the incidence and mortality of GC revealed by the study can contribute to defining new strategies for prevention, early detection, and, in general, GC management in the country.

Among the limitations, we acknowledge that we were unable to investigate specific epidemiologic characteristics of cardia and non-cardia GC and demonstrate differences. Another limitation is that as the cancer registry does not collect information about *H. pylori* infection among GC patients, we were not able to examine the effect of infection on the epidemiological features of GC. These limitations should be considered in future studies.

## Conclusions

The study demonstrates GC epidemiologic characteristics at the national level during the period 2015 and 2022. The disease remains a significant public health challenge in Georgia. GC Incidence and mortality rates in men are higher compared to women. No significant changes in the incidence of GC in males were detected, but in females, it decreased in 2022 compared to 2015. The mortality rates are still high, with only an unstable reduction. However, recently it has been more favorable in women, demonstrating decreasing trends in mortality after 2020. The majority of GC cases are diagnosed at an advanced stage, when, as usual, treatment options are limited and the prognosis is poor. Therefore, the implementation of prevention and early diagnosis strategies for GC is urgently needed in the country. Examining country-specific characteristics associated with *H. pylori* and other possible risk factors for GC should be considered in future studies to guide the implementation of appropriate primary prevention measures.
